# Hero Turned Villain: Identification of Components of the Sex Pheromone of the Tomato Bug, *Nesidiocoris tenuis*

**DOI:** 10.1007/s10886-021-01270-1

**Published:** 2021-04-12

**Authors:** David R. Hall, Steven J. Harte, Daniel P. Bray, Dudley I. Farman, Rob James, Celine X. Silva, Michelle T. Fountain

**Affiliations:** 1 Natural Resources Institute, University of Greenwich, Chatham Maritime, Kent, UK; 2 Thanet Earth Ltd, Barrow Man Road, Birchington, Kent, UK; 3 NIAB EMR, New Road, East Malling, Kent, UK

**Keywords:** GC-EAG, IPM, Mirid, 1-octanol, Octyl hexanoate, Hexyl octanoate

## Abstract

*Nesidiocoris tenuis* (Reuter) (Heteroptera: Miridae) is a tropical mirid bug used as a biocontrol agent in protected crops, including tomatoes. Although *N. tenuis* predates important insect pests, especially whitefly, it also causes damage by feeding on tomato plants when prey populations decline, resulting in significant economic losses for growers. The pest is now established in some all-year-round tomato crops in Europe and control measures involve the application of pesticides which are incompatible with current IPM programs. As part of future IPM strategies, the pheromone of *N. tenuis* was investigated. Volatile collections were made from groups and individuals of mated and unmated, females and males. In analyses of these collections by gas chromatography coupled with electroantennographic (EAG) recording from antennae of male bugs, two EAG-active components were detected and identified as 1-octanol and octyl hexanoate. Unlike other mirids, both male and female *N. tenuis* produced the two compounds, before and after mating, and both sexes gave EAG responses to both compounds. Furthermore, only octyl hexanoate was detected in whole body solvent washes from both sexes. These compounds are not related to the derivatives of 3-hydroxybutyrate esters found as pheromone components in other members of the Bryocrinae sub-family, and the latter could not be detected in volatiles from *N. tenuis* and did not elicit EAG responses. Nevertheless, experiments carried out in commercial glasshouses showed that traps baited with a blend of the synthetic pheromone components caught essentially only male *N. tenuis*, and significantly more than traps baited with octyl hexanoate alone. The latter caught significantly more *N. tenuis* than unbaited traps which generally caught very few bugs. Traps at plant height caught more *N. tenuis* males than traps 1 m above or at the base of the plants. The trap catches provided an indication of population levels of *N. tenuis* and were greatly reduced following an application of insecticide.

**Supplementary Information:**

The online version contains supplementary material available at 10.1007/s10886-021-01270-1.

## Introduction

*Nesidiocoris tenuis* (Reuter) (Heteroptera: Miridae) is an omnivorous mirid bug of tropical origin which is used as a biological control agent for protected crops in Mediterranean countries (Biondi et al. 2016; Pérez-Hedo et al. 2021). The bug predates a range of economically important crop pests such as whiteflies (family Aleyrodidae), aphids (superfamily Aphidoidea), thrips (order Thysanoptera), and moths (order Lepidoptera) (Kim et al. 2016). However, the use of *N. tenuis* as a biological control agent is controversial. Although this bug has a preference for prey, it can start feeding on the crop when prey is absent (Nakaishi et al. 2011; Pérez-Hedo and Urbaneja 2016; Sanchez 2008). Insertion of the stylet into the phloem to derive nutrients leads to brown necrotic rings around stems and petioles followed by the drying of flower stalks and flower abortion (Arnó et al. 2010; Calvo et al. 2009; Castañé et al. 2011; Raman and Sanjayan 1984). In some northern European countries *N. tenuis* has become invasive in glasshouses and the pest is threatening the 180 ha of tomatoes grown in the UK (Jacobson 2019).

In circumstances where *N. tenuis* needs controlling, traditional control options might not be suitable. Although the bug cannot complete development to adulthood if feeding exclusively on tomato plants, *Solanum lycopersicum* (Urbaneja et al. 2005), growers may resort to the use of chemical plant protection products (PPPs), incompatible with IPM programs, to prevent further crop damage. This can lead to a resurgence of whitefly populations and associated viruses, and disruption of other biocontrol systems such as use of predatory mites against spider mites. Moreover, a number of PPP’s have been shown to have lethal and sublethal effects on pollinators (Cloyd 2012; Feltham et al. 2014), and it is recommended that their use is avoided unless absolutely necessary (PAN Europe 2013).

Semiochemicals could provide tools for IPM-compatible management of *N. tenuis*. This species belongs to the Bryocorinae sub-family of mirids, and female-produced sex pheromones have been identified in several species of this sub-family. These include the cocoa mirids, *Distantiella theobroma* Dist. and *Sahlbergella singularis* Haglund (Mahob et al. 2011; Mahot et al. 2020; Sarfo 2013; Sarfo et al. 2018a, 2018b), and the aphidophagous mirid, *Macrolophus pygmaeus* Rambur (formerly *Macrolophus caliginosus* Wagner) (Gemeno et al. 2006). The components of these pheromones are derivatives of esters of 3-hydroxybutyric acid, rather different from those identified in mirid species from other subfamilies which are typically saturated or unsaturated, straight-chain esters or aldehydes (Zhang et al. 2020 and refs therein). For cocoa mirids, the synthetic pheromone has been used for monitoring (Mahob et al. 2011; Mahot et al. 2020; Sarfo 2013; Sarfo et al. 2018a) and mass trapping (Sarfo et al. 2018b).

To the best of our knowledge, the presence of a pheromone has not been demonstrated in *N. tenuis*. However, virgin females of the closely related *M. pygmaeus* attracted males (Gemeno et al. 2006). In this project we aimed to identify the female-produced sex pheromone of *N. tenuis* and demonstrate attraction of male *N. tenuis* to the synthesized pheromone in commercial glasshouses. This will provide a basis for development of pheromone traps for better detection of this invasive pest, for monitoring of pest numbers and more efficient use of control agents. The pheromone could also be used for control of *N. tenuis* by mass trapping or mating disruption and hence reduce or avoid the use of conventional pesticides against this pest on protected crops.

## Methods and Materials

### Chemicals

Unless otherwise stated, chemicals were purchased from SigmaAldrich (Gillingham, Dorset, UK) and were at least 99% pure. Hexyl (*R*)-3-hydroxybutyrate and hexyl (*R*)-3-[(*E*)-2-butenoyl]-butyrate) were synthesized as described previously (Padi et al. 2002), and hexyl (*R*)-3-acetoxybutyrate was prepared by acetylation of hexyl (*R*)-3-hydroxybutyrate with acetic anhydride and pyridine (Gemeno et al. 2006).

Octyl hexanoate used in the work described here was synthesized by reaction of 1-octanol with hexanoic acid in dichloromethane in the presence of N,N′-dicyclohexylcarbodiimide and a catalytic amount of 4-dimethylaminopyridine (Neises and Steglich 1978). The product was purified by flash chromatography on silica gel eluted with 2% diethyl ether in petroleum spirit (bp 40–60 °C) followed by Kugelrohr distillation (100 °C/0.06 mmHg) in 90% yield. Hexyl octanoate was prepared similarly by reaction of 1-hexanol and octanoic acid in 93% yield. Both compounds were characterized by their mass spectra and ^1^H and ^13^C NMR spectra (details in Supplementary Material). Octyl hexanoate has subsequently been prepared on molar scale in 95% yield by reaction of hexanoyl chloride with 1-octanol and pyridine in dichloromethane.

### Rearing of *Nesidiocoris tenuis*

Young tobacco (*Nicotiana spp*.), aubergine (*Solanum melongena*), tomato (*S. lycopersicum*), and cucumber (*Cucumis sativus*) were grown as host plants. Plants were sown separately in 11 × 11 × 11 cm pots containing standard compost and kept in a glasshouse at 26 ± 3 °C and 16:8 h L:D photoperiod. Organic dwarf beans used in bioassays and virgin adult cultures were obtained from a local supermarket (Sainsbury’s plc, London, UK).

*Nesidiocoris tenuis* adults were purchased from Bioline AgroSciences Ltd. (Nesiline) and a culture established in a quarantine facility at NIAB-EMR. Insects were reared in BugDorms (50 × 50 × 50 cm; MegaView Science, Taichung, Taiwan) on the host plants supplemented with sterile *Ephestia kuehniella* eggs (Nutrimac; Biobest, Ashford, UK), pollen and sugar solution (5% dextrose). Cultures were maintained at 20–26 °C and 55 to 65% relative humidity on a 16:8 h L:D photoperiod. BugDorm floors were lined with a layer of damp tissue to maintain humidity.

Initially, male and female adults were not separated for up to seven days before collections of volatiles were made. Mating was observed and nymphs were produced. To obtain virgin *N. tenuis* adults*,* third to fifth instar nymphs were housed individually in ventilated Perspex rearing boxes (8 × 6 × 14 cm). Nymphs were reared on sterile *E. kuehniella* eggs, a dwarf organic bean and 2.5 ml of sugar solution (5% dextrose) under the same conditions as BugDorm cultures. Virgin adults were kept in the individual rearing boxes and were 3–7 d old when collections of volatiles were made. Adults were sexed by observation of the abdomen with gentle pressure if necessary to show the paramere in males and ovipositor in females (Kim et al. 2016).

### Pheromone Collection

For collection of volatiles, virgin males and females were carefully placed in separate, silanized glass chambers (12 cm × 4 cm) with a glass frit at the upwind end (Hamilton Laboratory Glass, Margate, Kent, UK) containing an aubergine shoot or dwarf organic bean as food and maintained under the same conditions as the cultures above. Insects were left to settle in the chambers for at least 30 min prior to starting collection, and chambers containing food source only were used as controls. Air was drawn into the chamber with a vacuum pump (DA7C; Charles Austen, West Byfleet, UK) at 200 ml/min through a filter containing activated charcoal (20 cm × 2 cm, 10–18 mesh; Fisher Scientific, Loughborough, UK). Volatiles were collected on filters containing Porapak Q (50–80 mesh; 200 mg; Supelco, Gillingham, Dorset, UK) held between silanized glass wool plugs in a Pasteur pipette (4 mm i.d.). The Porapak was purified by Soxhlet extraction with chloroform and washing with dichloromethane before use. Volatiles were collected for 24 or 48 h.

Volatiles were first collected from groups of five (*N* = 3; aubergine as food) or 20 (*N* = 2) adult females and males which were presumed to be mated. Collections were then made from groups of 20 (*N* = 2) and individual (*N* = 9) virgin males and females. Collections were also made from chambers containing only a dwarf bean (*N* = 7). Volatiles were eluted from the Porapak with dichloromethane (1 ml; Pesticide Residue Grade), concentrated to approx. 100 μl under a gentle stream of nitrogen and stored at 4 °C before analysis.

Whole-body extracts were made by immersing individual *N. tenuis* adults in diethyl ether (0.5 ml; SLR grade containing butylated hydroxyl toluene, BHT, as antioxidant) for 10 min, then removing the ether, drying with a few grains of anhydrous magnesium sulfate and storing at 4 °C until analysis the same or following day. Extracts were made at approximately 10.00 h from individual females and males which were presumed to be mated (*N* = 7 and 8, respectively) and from virgin females and males (*N* = 2). Diethyl ether was used because potential compounds of interest such as (*E*)-4-oxo-2-hexenal are poorly soluble in hexane and are relatively stable in diethyl ether containing BHT (Fountain et al. 2014). An internal standard of decyl acetate (5 μg) was added for quantitative analysis.

### Analysis by Gas Chromatography Coupled to Electroantennographic Recording (GC-EAG)

GC-EAG Analyses were carried out on a HP6890 GC (Agilent Technologies, Manchester, UK) fitted with flame ionization detector (FID) and fused silica capillary columns (30 m × 0.32 mm i.d. × 0.25 μm film thickness) coated with DBWax and DB5 (Supelco). Injections onto the DBWax column were in splitless mode (220 °C), carrier gas was helium (2.4 ml/min) and the oven temperature was programmed from 50 °C for 2 min and then at 20 °C/min. to 250 °C for 3 min. The effluents of the two columns were combined with a glass push-fit Y-tube connector (Agilent Technologies) connected to a second Y-tube connector with deactivated fused silica tubing (10 cm × 0.32 mm i.d.). One arm of this connector was connected with deactivated fused silica tubing (50 cm × 0.32 mm i.d.) to the FID (250 °C) and the other to an equal length of deactivated silica tubing passing through a heated transfer line (250 °C; Syntech, Hilversum, The Netherlands, now Kirchzarten, Germany) into a glass tube (4 mm i.d.) through which air passed (500 ml/min) over the EAG preparation. Both the FID and EAG signals were collected and analyzed with EZChrom software (Elite v3.0; Agilent Technologies).

For EAG recordings, adult *N. tenuis* were anesthetized using carbon dioxide, and the head and one antenna removed under a dissecting microscope with a razor blade. A borosilicate glass capillary electrode (ID 0.86 mm, Warner Instruments, Hamden, CT 06514), pulled to a fine tip and filled with Beadle-Ephrussi Ringer containing 1% polyvinylpyrrolidine as electrolyte, was inserted into the back of the head. The electrode and head were then mounted onto a silver wire held within an electrode holder connected to the earth probe of a portable EAG amplifier (INR-2, Syntech). A similar electrode mounted onto the ×10 recording preamplifier was then brought into contact with the distal tip of the antenna. Straightening the antenna between the electrodes reduced background noise from the preparation.

### Analysis by Gas Chromatography Coupled to Mass Spectrometry (GC-MS)

GC-MS Analyses were carried out on a CP3500 GC coupled to a CP2200 Saturn Ion Trap Detector (Varian, now Agilent Technologies). The GC was fitted with fused silica capillary columns (30 m × 0.25 mm i.d. × 0.25 μm film thickness) coated with DBWax (Supelco) and VF5 (Varian) with a switching device to select the column used. Carrier gas was helium (1 ml/min) and manual injections (1 μl) were made in splitless mode (220 °C), with oven temperature programmed from 40 °C for 2 min then at 10 °C/min to 240 °C. Compounds were identified according to their mass spectrum, retention index relative to retention times of *n*-alkanes, and co-chromatography with authentic compounds.

### Analysis by Gas Chromatography with Flame Ionization Detection (GC-FID)

GC-FID Analyses were carried out on HP6850 instruments (Agilent) fitted with fused silica capillary columns (30 m × 0.32 mm i.d. × 0.25 μm film thickness) coated with polar DBWax (Supelco) or non-polar HP5 (Agilent) and FID detectors (250 °C). Carrier gas was helium (2.4 ml/min), injection was splitless (220 °C and 250 °C, respectively) and the oven temperature was programmed from 50 °C for 2 min then at 10 °C/min to 250 °C. Data were collected and analyzed with EZChrom software (Elite v3.0; Agilent).

### Controlled Release Dispensers

Two types of controlled-release dispenser for the pheromone components were investigated. The first were opaque, polypropylene pipette tips (1 ml; Fisher Scientific) with a 0.2 mm tip aperture, sealed with a Teflon-lined crimp seal (11 mm; Chromacol, Welwyn Garden City, UK) as developed for the pheromone of *Lygus* bugs by Fountain et al. (2014). The second were sealed low-density polyethylene vials (22 mm × 8 mm × 1.5 mm thick; Just Plastics, London, UK).

A blend of equal weights of 1-octanol and octyl hexanoate was formulated as the neat material (25 μl) or a 10% solution in sunflower oil (100 μl) and applied to cellulose acetate cigarette filters (14 mm × 6 mm; Swan, High Wycombe, Buckinghamshire, UK) in the dispensers. Two vials for each type of dispenser and loading were maintained in a wind tunnel (27 °C and 8 km/h windspeed) and release rates were measured by periodic trapping of volatiles on Porapak followed by quantitative GC-FID analysis using decyl acetate (5 μg) as internal standard, as described for collection of pheromone from insects.

### Field Trials in Commercial Glasshouses

Two field trapping experiments were carried out on tomato in commercial glasshouses in Kent, UK. Varieties included Piccolo, Summer Sun, and Sun Stream. Temperature and other meteorological data were recorded every five minutes; during the period of the trapping experiments the mean temperature was 17.6 °C, with maximum 25.5 °C and minimum 13.0 °C. Traps were yellow “Drystick” (dry glue) sticky traps (25 cm × 10 cm; Koppert, Ashford, UK) with the lure attached to the center by a twist-tie. In preliminary tests, white sticky traps were used with standard polybutene glue, but laboratory and field studies indicated the bugs could escape from this.

In the first trial (19 December 2019–09 January 2020) there were 10 blocks of three treatments, with one replicate of each treatment per block. Treatments formulated on cigarette filters in polyethylene vials were (A) a 1:1 blend of neat 1-octanol and octyl hexanoate (25 μl), (B) neat octyl hexanoate (12.5 μl), (C) blank control of a cigarette filter only. In eight blocks traps were positioned at 6 m above ground, just above the crop canopy, and in the other two blocks traps were positioned at 1 m, within the crop canopy. Traps were spaced >10 m apart, with blocks in a continuous row, to minimize interference between treatments. Trap catches were recorded weekly on 27 December 2019, 3 January, and 9 January 2020. However, the glasshouse was treated with insecticide (Pyrethrum 5EC) on 4 January 2020 because of the high catches of *N. tenuis*. Catches were low in the following week and only data from the first two weeks were used in analyses.

In the second experiment (10 January – 30 January 2020), the three treatments were (A) and (C) as above, and treatment (D) containing a 1:1 blend of 1-octanol and octyl hexanoate as a 10% solution in sunflower oil, resulting in an approximately 10-fold reduction in release rate relative to that from (A). There were 15 blocks with one replicate of each treatment per block. Eight blocks were positioned immediately above the crop canopy, 4 blocks 1 m above the crop canopy, and 3 blocks just above the base of the plants. Catches were recorded on 18, 25 and 30 January 2020.

In all tests the same sticky trap was used throughout each experiment. *N. tenuis* captured were counted and sexed on the trap and circled with a marker pen to differentiate them from new catches the following week. Sticky traps were collected from the glasshouse at the end of experiment and sexing was confirmed under a microscope by dissecting open the posterior abdomen and identifying the paramere in the males and ovipositor in the females (Kim et al. 2016).

For each experiment, catches of males in each trap were summed over the course of the experiment and transformed to ln(x + 1) to normalize variance. Trap catch was entered as the dependent variable in a mixed model, with treatment and trap height as fixed factors, and block entered as a random factor (Bates et al. 2015). A treatment * trap height interaction was included to test whether numbers of insects caught by each treatment varied with height. Statistical significance of fixed factors was determined through χ^2^ tests of changes in residual deviance following deletion from the model (Crawley 2013). Post-hoc comparisons between catches made by different treatments and at different traps were made using Tukey’s tests on estimated marginal means (Lenth 2019). All analyses were performer in R Version 3.3.3.

## Results

### Pheromone Identification

**Fig. 1:**
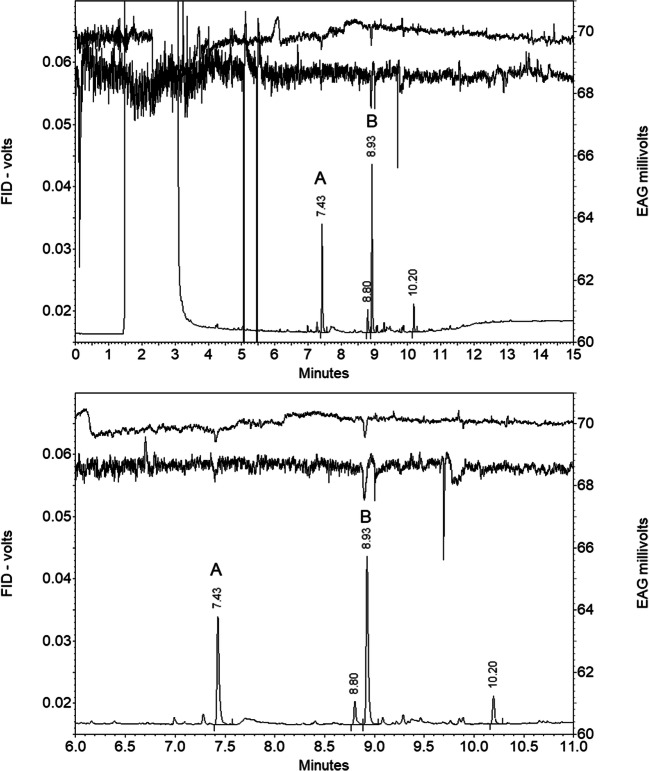
GC-EAG analyses of volatile collection from virgin female *Nesidiocoris tenuis* with male antennal EAG preparation on polar GC column showing EAG responses to components (A) at 7.43 min and (B) at 8.93 min; lower trace is expansion of upper (other compounds 7.00 min 2-ethylhexanol; 7.28 min benzaldehyde; 8.80 min methyl salicylate; 9.30 and 10.20 min Porapak impurities)

Initial GC-EAG analyses of volatile collections from mated female *N. tenuis* showed two peaks (A and B) eliciting consistent EAG responses from antennae of mated male *N. tenuis.* The same result was obtained in analyses of volatiles from a single unmated female with antennae of unmated males (Fig. 1).

**Fig. 2:**
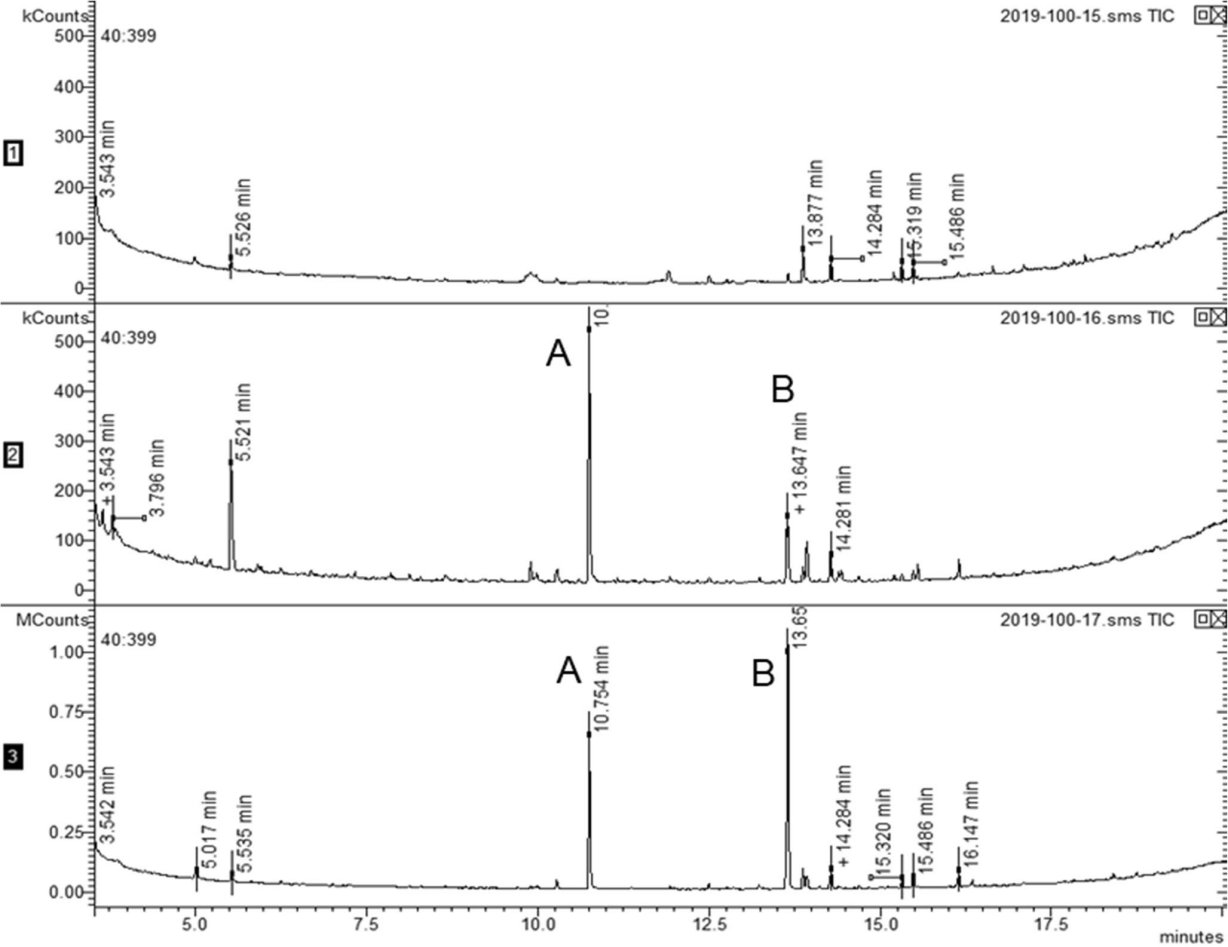
GC-MS analyses on polar GC column of volatile collections from green bean (upper), single virgin male *Nesidiocoris tenuis* (middle), and single virgin female *N. tenuis*, showing peaks A and B corresponding to EAG responses

**Fig. 3:**
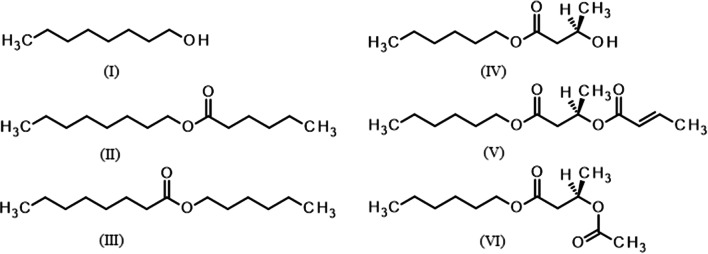
Structures of pheromone components and analogs: (I) 1-octanol (component A); (II) octyl hexanoate (component B); (III) hexyl octanoate; (IV) hexyl (*R*)-3-hydroxybutyrate; (V) hexyl (*R*)-3-[(*E*)-2-butenoyl]-butyrate; (VI) hexyl (*R*)-3-acetoxybutyrate

In analyses of these collections by GC-MS, the two EAG-active components (A) and (B) were present in volatiles from both mated and unmated female *N. tenuis* and in volatiles from both mated and unmated males, but not detectable in volatiles from green beans used as food source during the volatile collections (Fig. 2). The mass spectra indicated these two peaks corresponded to 1-octanol and octyl hexanoate, respectively (structures (I) and (II) in Fig. 3), and the corresponding synthetic compounds had identical mass spectra and retention times on both polar and non-polar GC columns (Table 1).

**Table 1:** GC Retention Indices of compounds on polar and non-polar GC columns (relative to the retention times of *n*-alkanes)

	Retention Index		
	Polar		Non-polar
Compound	GC-EAG	GC-MS	GC-MS
EAG-active compound (A)	1557	1566	1074
EAG Active compound (B)	1815	1820	1584
1-octanol (I)	1557	1566	1074
octyl hexanoate (II)	1815	1820	1584
hexyl octanoate (III)	1815	1820	1584
hexyl (*R*)-3-hydroxybutyrate (IV);	1912	1921	1338
hexyl (*R*)-3-[(*E*)-2-butenoyl]-butyrate (V)	2232	2242	1709
hexyl (*R*)-3-acetoxy-butyrate (VI)	1942	1957	1487

Detailed examination of the GC-MS traces failed to detect the cocoa mirid pheromone components, hexyl (*R*)-3-hydroxybutyrate (IV in Fig. 3) and hexyl (*R*)-3-[(*E*)-2-butenoyl]-butyrate (V), or the proposed pheromone of *M. pygmaeus*, hexyl (*R*)-3-acetoxybutyrate (VI) (Table 1).

**Fig. 4:**
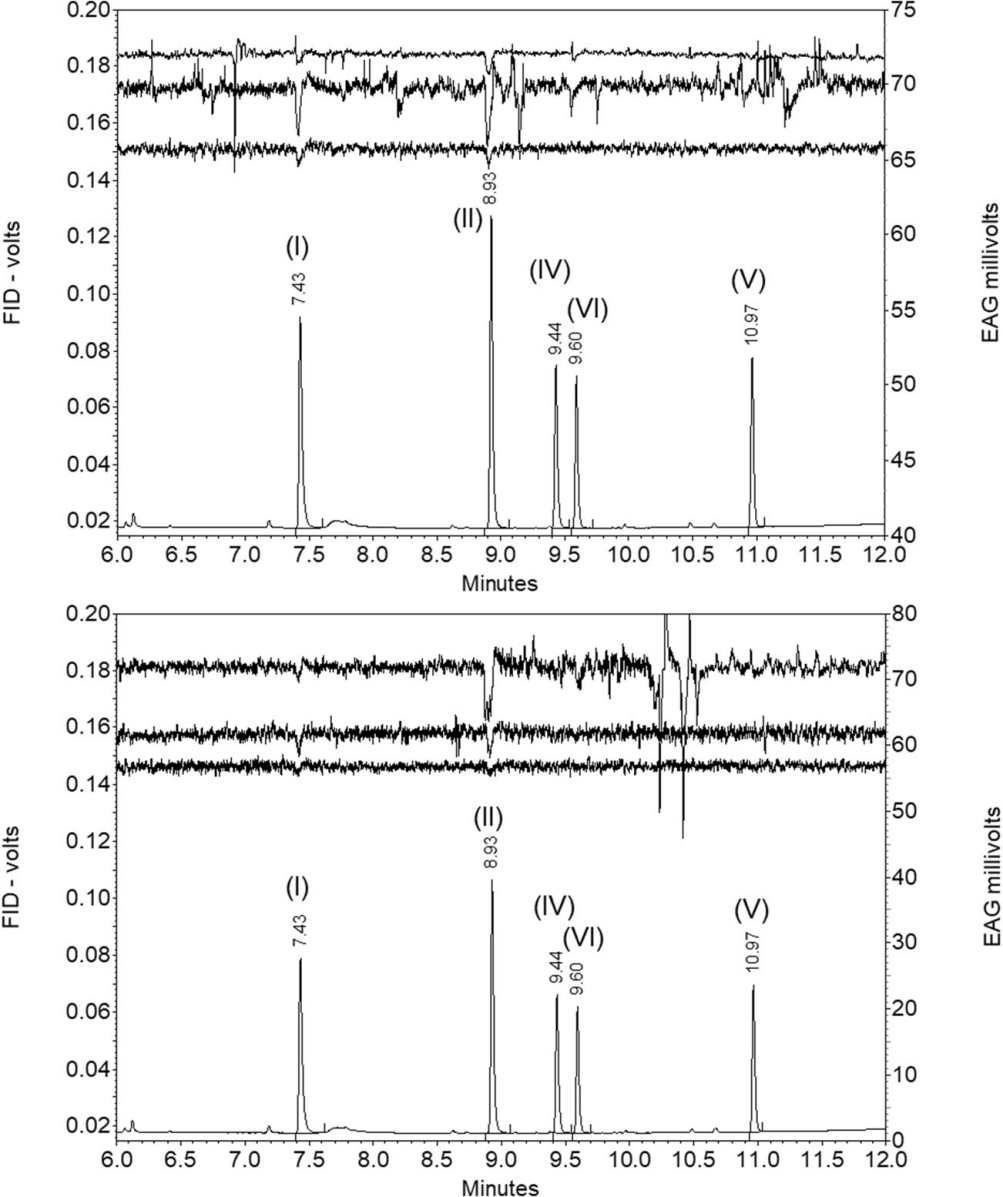
GC-EAG analyses of synthetic compounds (20 ng injected) with male (upper) or female (lower) *Nesidiocoris tenuis* antennal EAG preparation on polar GC column showing EAG responses to 1-octanol (I, component A) at 7.43 min and octyl hexanoate (II, compound B) at 8.93 min and also possibly at to hexyl (*R*)-3-acetoxybutyrate (VI) at 9.60 min but not to hexyl (*R*)-3-hydroxybutyrate (IV) at 9.44 min; or hexyl (*R*)-3-[(*E*)-2-butenoyl]-butyrate (V) at 10.97 min

In GC-EAG analyses of synthetic compounds, 1-octanol (I) and octyl hexanoate (II) elicited EAG responses from antennae of both male and female *N. tenuis*. No EAG response was observed to the cocoa mirid pheromone components, hexyl (*R*)-3-hydroxybutyrate (IV) and hexyl (*R*)-3-[(*E*)-2-butenoyl]-butyrate) (V), but a small response was sometimes observed to the proposed pheromone component of *M. pygmaeus*, hexyl (*R*)-3-acetoxybutyrate (VI) (Fig. 4).

The analogue of octyl hexanoate, hexyl octanoate (III) had identical retention times to octyl hexanoate on both polar and non-polar GC columns (Table 1), but clearly different mass spectrum. The mass spectrum of octyl hexanoate shows the anticipated diagnostic base peak at m/z 117, corresponding to protonated hexanoic acid (C_5_H_11_COOH_2_^+^), while that of hexyl octanoate shows a similarly diagnostic base peak at m/z 145 corresponding to protonated octanoic acid (C_7_H_15_COOH_2_^+^) (Supplementary Material, Fig. S1). In GC-EAG analyses, the analogue, hexyl octanoate, elicited a consistent EAG response from the antennae of male *N. tenuis* (Supplementary Material, Fig. S2).

### Quantification of Pheromone Components

Of the 31 collections of volatiles made from *N. tenuis* adults, only two collections – one virgin female and one virgin male – contained no detectable pheromone components. The rest contained detectable amounts of both pheromone component (A), 1-octanol (I), and pheromone component (B), octyl hexanoate (II), as shown in Table 2.

**Table 2:** Amounts and relative ratio of pheromone components in collections of volatiles and whole-body extracts from *Nesidiocoris tenuis* (analysis GC-FID on non-polar GC column against external standard for volatiles, internal standard 5 μg decyl acetate for extracts)

	Mean (SE or range)		
Source	Component A octanol (I)	Component B octyl hexanoate (II)	Ratio octyl hexanoate/octanol
Volatile Collections (ng/h/insect)			
5–20 mated female (*N* = 5)	1.3 (0.2)	0.8 (0.4)	0.6 (0.2)
5–20 mated male (*N* = 5)	3.7 (0.5)	0.3 (0.04)	0.1 (0.01)
20 virgin female (*N* = 2)	4.6 (2.3–6.9)	3.3 (2.7–3.9)	0.9 (0.6–1.2)
20 virgin male (*N* = 2)	3.7 (3.0–4.4)	0.4 (0.4–0.4)	0.1 (0.1–0.1)
single virgin female (*N* = 8)	2.9 (1.1)	2.5 (0.8)	1.1 (0.3)
single virgin male (*N* = 5)	0.7 (0.1)	0.6 (0.2)	1.1 (0.6)
Extracts (ng/insect)			
virgin female (*N* = 2)		1826 (1292-2360)	
virgin male (*N* = 2)		1712 (993–2432)	
mated female (*N* = 6)		750 (197)	
mated male (*N* = 6)		821 (215)	

In collections from groups of 5–20 insects, the ratio of octyl hexanoate/octanol was higher in those from females than in those from males for both mated (0.6 ± 0.2 SE and 0.1 ± 0.01 respectively), and unmated (0.9 and 0.1 respectively) insects. For single, unmated insects, the ratios were the same (1.1) although more of both components was produced by females than males (Table 2).

Unexpectedly, whole body extracts of mated and unmated males and females contained only octyl hexanoate, and 1-octanol could not be detected (<<0.01%). For both females and males, amounts in unmated insects seemed to be higher than in mated, although only two unmated insects were extracted (Table 2).

### Controlled Release Dispensers

**Fig. 5:**
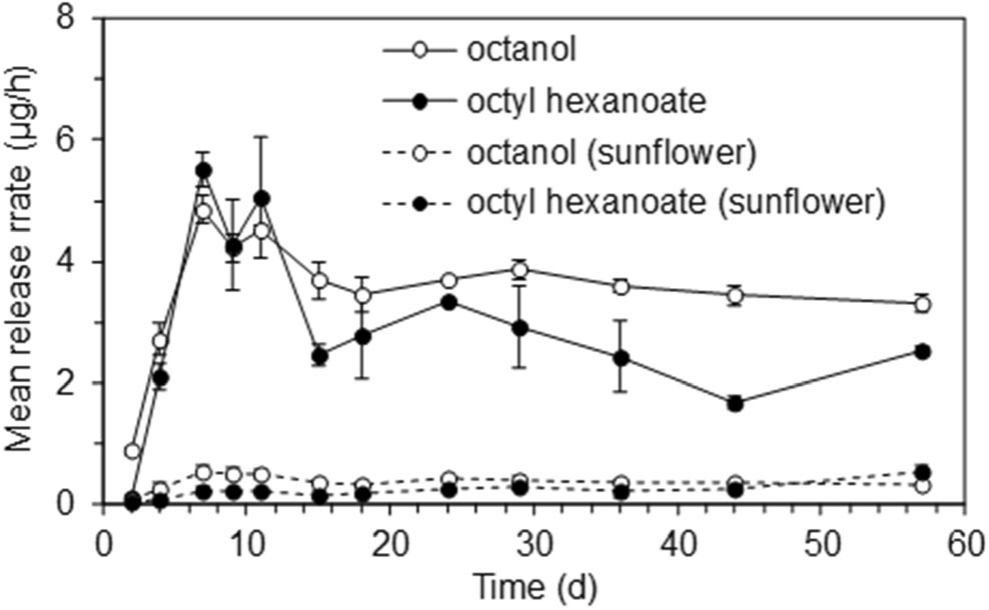
Mean release rates (*N* = 2; bars indicate range) of 1-octanol and octyl hexanoate from polyethylene vial dispensers containing a 1:1 blend of the components as a 10% solution in sunflower oil (100 μl) or neat (25 μl); dispensers maintained in windtunnel at 27 °C and 8 km/h windspeed

Pipette tips and sealed polyethylene vials were investigated as controlled release dispensers for the synthetic pheromone components, using a 1:1 blend of 1-octanol and octyl hexanoate either neat or as a 10% solution in sunflower oil. Release of octyl hexanoate from the pipette tips was negligible and these were not investigated further. Polyethylene vials released both components for over six weeks at 27 °C and 8 km/h windspeed (Fig. 5). The release rates for the neat material were approximately ten times those for the solution in sunflower oil. The ratio of octyl hexanoate/ octanol during the first month of exposure was approximately 0.8 for the neat material and 0.5 for the sunflower solution.

### Field Trapping

**Fig. 6:**
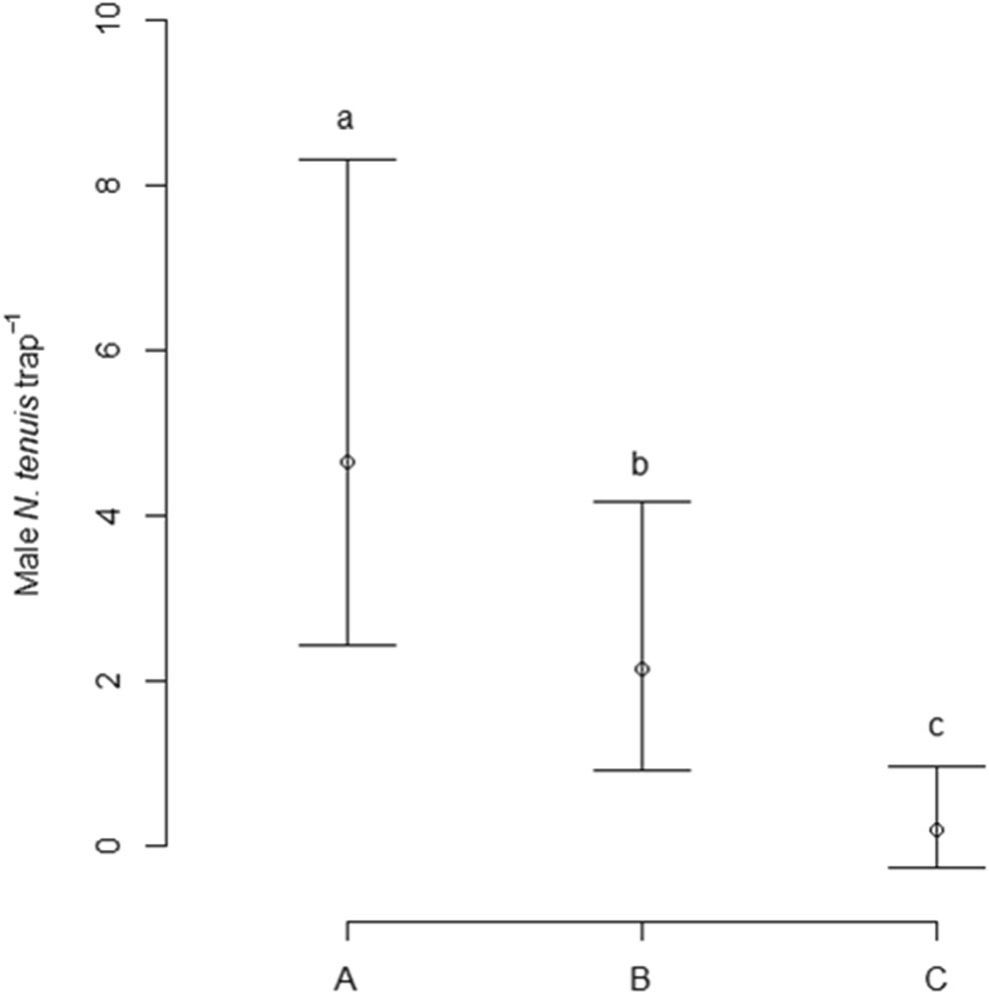
Estimated mean catches of *Nesidiocoris tenuis* per trap per week (± 95% confidence intervals) in commercial glass house in Experiment 1 (19/12/2019–3/1/2020); (A) neat 1-octanol + octyl hexanoate; (B) octyl hexanoate; (C) unbaited control, *N* = 10; means labelled with different lower case letters are significantly different form one another (Tukey’s post hoc test, *P* < 0.05); means are averaged over treatments at different trap heights

Two trapping tests were carried out in commercial greenhouses. In the first trial, a significant effect of treatment was found on numbers of male *N. tenuis* caught (χ^2^ = 21.8, df = 2, *P* < 0.001). Traps baited with the two-component blend of 1-octanol and octyl hexanoate (Treatment A) caught significantly more male *N. tenuis* than traps baited with octyl hexanoate alone (Treatment B, Tukey’s test, *P* < 0.05). Treatments A and B caught more male *N. tenuis* than the blank control (Treatment C, Tukey’s test, *P* < 0.05) (Fig. 6). No evidence was found for an effect of trap height (6 m or 1 m above ground; χ^2^ = 0.14, df = 1, *P* = 0.71) or an interaction between trap height and treatment (χ^2^ = 2.7, df = 2, *P* = 0.26) on trap catches. Six female *N. tenuis* were captured across the experiment (Treatment A: two females, B: two females, C: two females). In this experiment, the glasshouse was treated with insecticide after two weeks and the total catch of male *N. tenuis* dropped from 56 and 58 in the first two weeks, respectively, to 11 in the third week. Only catches from the first two weeks were used in the analyses.

**Fig. 7:**
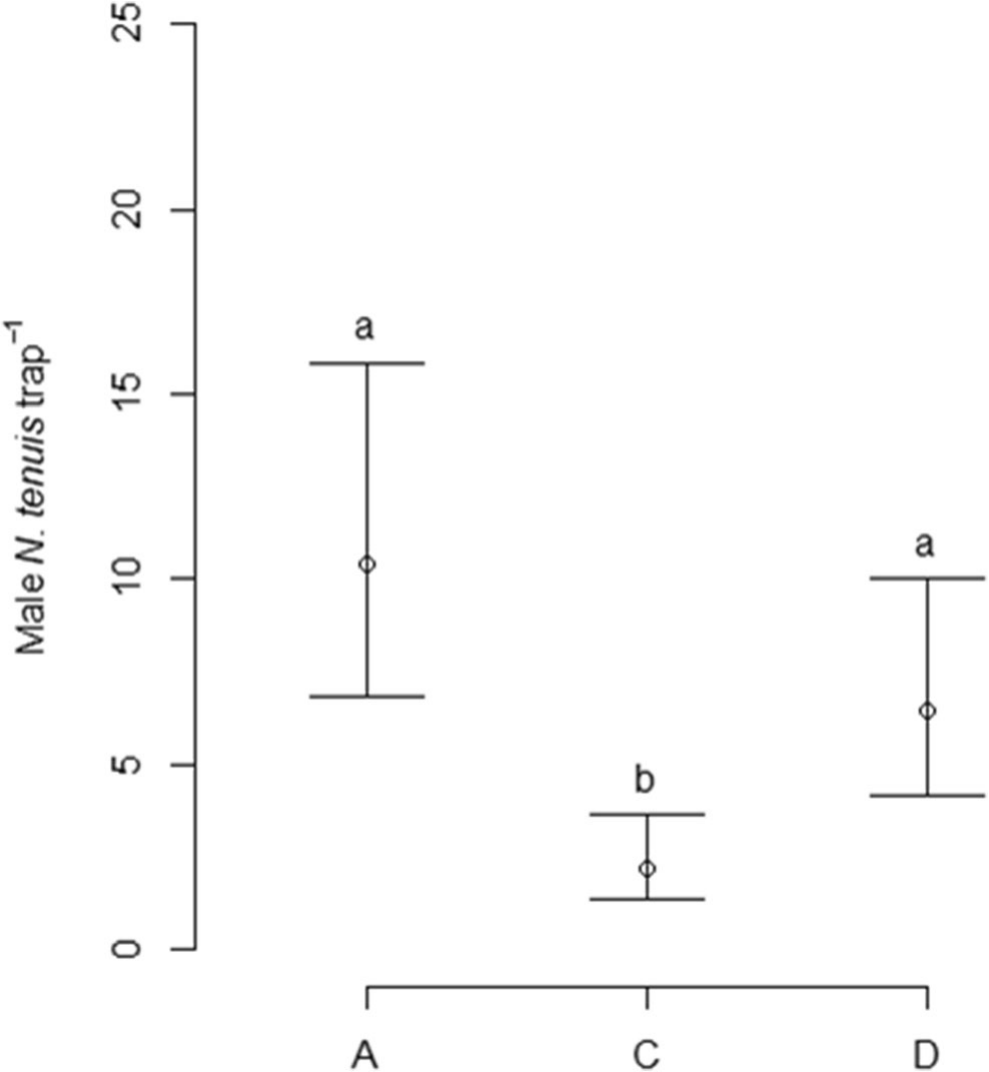
Estimated mean catches of *Nesidiocoris tenuis* males per trap per week (± 95% confidence intervals) in a commercial glasshouse during Experiment 2 (10–30/1/2020; (A) neat 1-octanol + octyl hexanoate; (D) 1-octanol + octyl hexanoate 10% in sunflower oil; (C) unbaited control *N* = 15; means with different letters are significantly different (Tukey’s post hoc test, *P* < 0.05); means are averaged over treatments at different trap heights

**Fig. 8:**
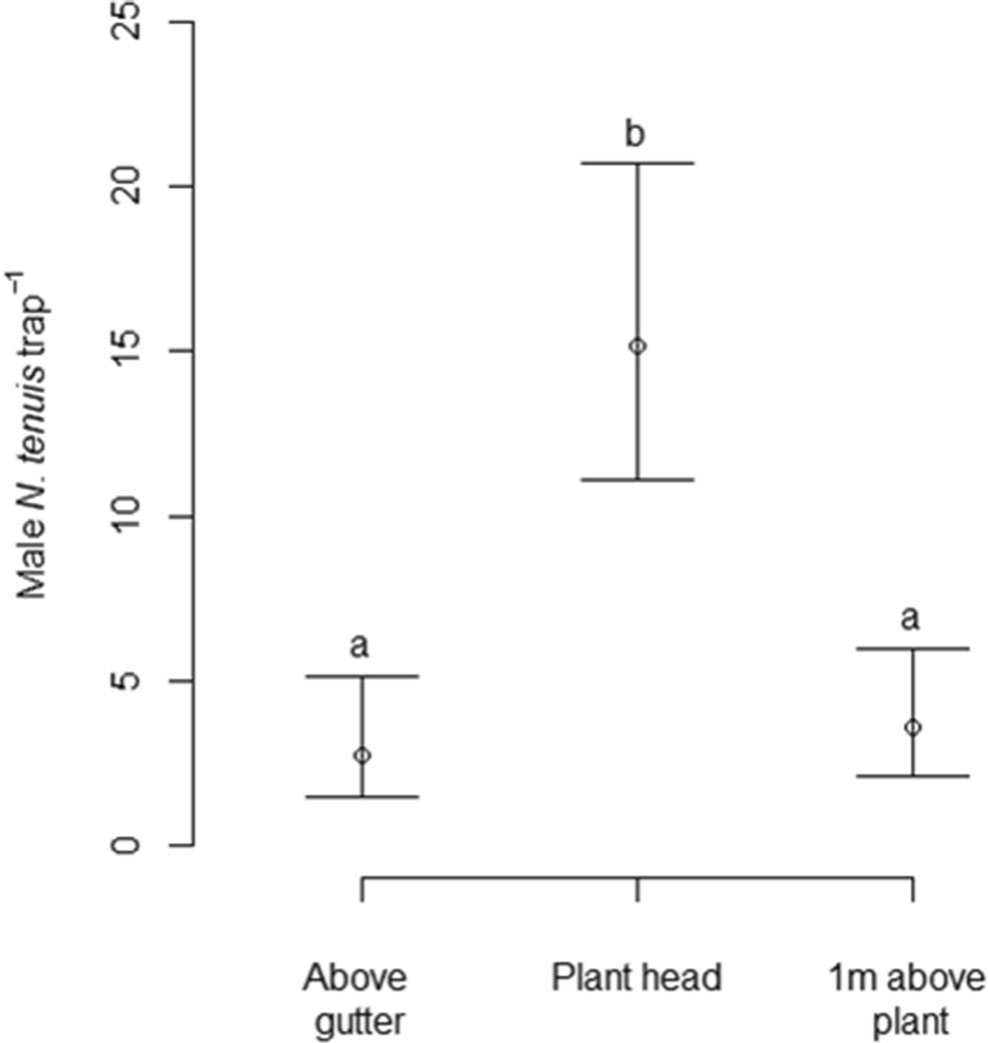
Mean catches of *Nesidiocoris tenuis* males in traps at different heights during Experiment 2 (10–30/1/2020; (A) neat 1-octanol + octyl hexanoate; (D) 1-octanol + octyl hexanoate 10% in sunflower oil; (C) unbaited control; plant base (*N* = 3), plant head (*N* = 8), 1 m above plant (*N* = 4); means with different letters are significantly different (Tukey’s post hoc test, *P* < 0.05)

In the second experiment, significant effects of both treatment (χ^2^ = 33.0, df = 2, *P* < 0.001) and trap height (χ^2^ = 12.3, df = 2, *P* < 0.01) were found on catches of male *N. tenuis*. However, there was no evidence of a Treatment * Trap height interaction on catches (χ^2^ = 2.5, df = 4, *P* = 0.65). Averaged over trap height, Treatments A and D caught significantly more male *N. tenuis* than control treatment C (Tukey’s post hoc test on estimated marginal means, *P* < 0.05), but lures with the higher release rate (A) did not catch significantly more bugs than those with a ten-fold lower release rate (D) (Fig. 7). Averaged over treatments, significantly more male *N. tenuis* were caught at the top of the plant compared to 1 m above or at the plant base (Tukey’s test, *P* < 0.05) (Fig. 8). Three female *N. tenuis* were caught in traps baited with treatment A, three with treatment C and seven with treatment D. Eight of the females were caught on traps positioned at the tops of the plants, three were caught 1 m above the plant, and 2 were caught at the plant base.

## Discussion

Two components of the sex pheromone produced by adult female *N. tenuis* have been identified as 1-octanol and octyl hexanoate. In trapping tests carried out in commercial tomato glasshouses, traps baited with octyl hexanoate alone caught significantly more male *N. tenuis* than unbaited traps, but significantly less than traps baited with a 1:1 blend of the two components.

### Pheromone Identification

Octyl hexanoate was reported as a minor component (5–7% of major component, hexyl hexanoate) in volatiles produced by females of another mirid bug, *Trigonotylus caelestialium* Kirkaldy, but was not important for attraction of males (Kakizaki and Sugie 2001). It is also reported to be involved in the chemical ecology of various stingless bees, e.g. of the genus *Trigona* (Hymenoptera; Apidae). For example, it is a component of a trail pheromone and labial gland secretion (Jarau et al. 2010), of a recruitment pheromone (Lichtenberg et al. 2011), and of the cephalic secretion (Francke et al. 2000). The isomer, hexyl octanoate has also been reported to be present in these stingless bee secretions, but 1-octanol has not previously been reported as an insect semiochemical. This compound was shown to be produced by female *Macrolophus pygmaeus,* but its role was not established (Gemeno et al. 2006).

*Nesidiocoris tenuis* belongs to the Bryocorinae sub-family of mirids, and female-produced sex pheromones have been identified in several species of this sub-family. These include the cocoa mirids, *Distantiella theobroma* (Dist.) and *Sahlbergella singularis* Haglund, (Mahob et al. 2011; Mahot et al. 2020; Sarfo 2013; Sarfo et al. 2018a, 2018b), and the aphidophagous mirid, *M. pygmaeus* (Gemeno et al. 2006). The components of these pheromones are derivatives of esters of 3-hydroxybutyric acid, and rather different from those identified in mirid species from other subfamilies which are typically saturated or unsaturated, straight-chain esters or aldehydes (Zhang et al. 2020 and refs therein). The pheromone components identified in *N. tenuis*, 1-octanol and octyl hexanoate, are more like the latter than the former, and the cocoa mirid and *M. pygmaeus* pheromone components could not be detected in volatiles or extracts from female or male *N. tenuis*. Furthermore, the synthetic compounds did not elicit a consistent EAG response from antennae of male *N. tenuis*.

It was also unexpected that both pheromone components are released by both mated and unmated male and female *N. tenuis* when in groups or as isolated individuals. For many species of mirid, the same compounds typically serve as pheromone components and also as defensive compounds (Aldrich 1988; Fountain et al. 2014; Staddon 1986). Thus, in four *Lygus* species, Fountain et al. (2014) showed that three compounds could be extracted from both females and males, and when volatiles were collected from groups of insects, all three compounds were produced in a similar ratio from females and males. However, when volatiles were collected from individual, undisturbed virgin insects, the three compounds were only produced by females and in a species-specific ratio that constituted the sex pheromone and was different from that produced by groups of insects.

Collections from *N. tenuis* were rather different. In those from insects in groups, the ratio of octyl hexanoate/1-octanol was higher from females than males whether mated (0.6 and 0.1 respectively) or unmated (0.9 and 0.1 respectively). In collections from individual insects the ratios from females and males were essentially the same (1.1), although results from individuals were very variable at 0.4–3.0 in females and 0.2–3.4 in males.

Even more surprising was the observation that, although the two compounds were found in volatiles from mated and unmated *N. tenuis* groups and individuals of males or females, whole body extracts in diethyl ether of mated and unmated males or females contained only octyl hexanoate with no 1-octanol detectable. This result is difficult to explain unless the 1-octanol is contained in some gland not extracted by the solvent. Significant crushing of the bodies was avoided in order not to extract extraneous body materials that could damage the GC column used for analysis, but light compression of the bodies failed to change the result. The same technique has been used on several other mirid species and gave extraction of all pheromone components. One possible factor is that the extracts here were all made at a similar time in the diurnal cycle. Volatile collections were made for complete 24 h or 48 h periods, and analyses of whole body extracts made at different times could provide different results.

It is also possible that production of 1-octanol was induced by herbivore feeding on the green beans used as food for the bugs during collections of volatiles. However, in our studies, no 1-octanol was detected in collections from intact beans in the absence of insects, or from beans punctured with a dissecting needle. Both 1-octanol and octyl hexanoate were obtained in initial collections from bugs on aubergines in similar absolute and relative amounts to those obtained in collections from bugs on green beans. Induction of production of volatiles by *N. tenuis* has been reported for tomatoes (Pérez-Hedo et al. 2018) and sweet peppers (Bouagga et al. 2018), but 1-octanol was not reported in these studies. Both 1-octanol and octyl acetate were detected in whole body extracts of female *M. pygmaeus,* along with proposed pheromone components hexyl and octyl 3-acetoxybutanoate (Gemeno et al. 2006). In collections of volatiles from *M. pygmaeus* with or without green beans, octyl acetate was the major component with much smaller amounts of the other compounds detected (Gemeno et al. 2006). Thus it is concluded that 1-octanol is most likely produced by *N. tenuis* adults.

### Field Trapping

In the initial studies of volatiles released by mated insects, the amount of 1-octanol was relatively higher than that of octyl hexanoate. Nevertheless, it was thought that 1-octanol was possibly a biosynthetic precursor and that octyl hexanoate was the “major” pheromone component. Thus, in the first trapping experiment, because of the limited resources available, only lures containing the two-component blend or octyl hexanoate alone were tested. Traps baited with the latter did catch more male *N. tenuis* than unbaited traps, but those baited with the two-component blend caught significantly more. It was not possible to test the 1-octanol alone during the short timeframe of this project, but obviously this should be done in future work.

Polyethylene vials provided convenient dispensing systems for the two pheromone components. The release rate could be adjusted by diluting the components in an involatile diluent such as sunflower oil, and the ratio in the blend released could be modified by changing the ratio in the blend loaded. The blend released with the 1:1 blend loading used in the trapping tests approximated to the mean ratio produced by virgin females, but the latter was very variable and there is scope to optimize the blend and release rate from the vials.

In the first trapping experiment, lures were used with release rates of approximately 4 μg/h. This is three orders of magnitude greater than the amounts recorded from individual *N. tenuis* females. Heteropteran bugs often use similar compounds as pheromones and as defense compounds with the latter produced at much higher release rates than the former (Aldrich 1988; Fountain et al. 2014; Staddon 1986). Thus, in the second trapping experiment lures with a ten-fold lower release rate were tested. Catches of *N. tenuis* males with the lower release rate were lower than those with the higher rate, although not significantly so in this experiment because of the high variability in catches. However, it would seem that use of very low release rates is not necessary with *N. tenuis*.

In the second trapping experiment, on two occasions the unbaited traps caught as many male *N. tenuis* as the baited traps. Occasional high catches have been observed in routine use of unbaited monitoring traps (Janos Domsodi, pers. comm), and this has been attributed to capture of a virgin female bug on the trap which emitted pheromone and attracted males. In the present trial, the lures were checked at the end of the experiment and shown to be both correctly labelled and free of contamination. A female was found on the trap in one case, but not in the other, although the bugs are known to be capable of escaping from the glue on occasions.

Intraspecific attraction in *N. tenuis* has not been studied with live insects. Even though this study showed males produce the same chemicals as females under laboratory conditions, it is not known whether males attract males. Catches of insects in unbaited traps in the trapping trials here were very low but not zero, so if males were attracting males then it would have been expected that catches on the unbaited traps might have been higher.

Catches in individual traps were very variable. There was some suggestion that certain traps caught higher numbers than others, but this was not always consistent from week to week. This may reflect fluctuations in local populations, and plant growth stage. The effect of an insecticide application during the first trapping experiment was accurately reflected in a marked reduction in trap catches. However, catches in pheromone traps are influenced by a number of factors and further research is required to understand the reasons for this. For example, at high populations trap catches may be reduced due to female insects competing with the traps, as has been found in the mirid bug *Lygus hesperus* L. (Hall, Millar and Daane, unpublished).

These results provide the basis for development of monitoring traps for detection and surveillance of *N. tenuis,* although further research is required to correlate trap catches with damage thresholds in crops. Given the ready availability of the pheromone, the possibilities for using it in control of *N. tenuis* through mass trapping or mating disruption could be investigated, as well as the potential of the pheromone isomer, hexyl octanoate, as an inhibitor or repellent.

## Supplementary Information

ESM 1 (DOCX 112 kb)

## References

[CR1] Aldrich JR (1988). Chemical ecology of the Heteroptera. Annu Rev Entomol.

[CR2] Arnó J, Castañé C, Riudavets J, Gabarra R (2010). Risk of damage to tomato crops by the generalist zoophytophagous predator *Nesidiocoris tenuis* (Reuter) (Hemiptera: Miridae). B Entomol Res.

[CR3] Bates D, Maechler M, Bolker B, Walker S (2015). Fitting linear mixed-effects models using lme4. J Statistical Software.

[CR4] Biondi A, Zappalà L, Di Mauro A (2016). Can alternative host plant and prey affect phytophagy and biological control by the zoophytophagous mirid *Nesidiocoris tenuis*?. Biocontrol.

[CR5] Bouagga S, Urbaneja A, Rambla JL, Flors V, Granell A, Josep A, Jaques JA, Pérez-Hedoa M (2018). Zoophytophagous mirids provide pest control by inducing direct defences, antixenosis and attraction to parasitoids in sweet pepper plants. Pest Manag Sci.

[CR6] Calvo J, Bolckmans K, Stansly PA, Urbaneja A (2009). Predation by *Nesidiocoris tenuis* on *Bemisia tabaci* and injury to tomato. Biocontrol.

[CR7] Castañé C, Arnó J, Gabarra R, Alomar O (2011). Plant damage to vegetable crops by zoophytophagous mirid predators. Biol Control.

[CR8] Cloyd RA (2012). Indirect effects of pesticides on natural enemies. Pesticides—advances in chemical and botanical pesticides. RP Soundararajan Ed, IntechOpen, London, UK. p.382

[CR9] Crawley MJ (2013) The R book, Second Edition. John Wiley and Sons Ltd, UK

[CR10] Feltham H, Park K, Goulson D (2014). Field realistic doses of pesticide imidacloprid reduce bumblebee pollen foraging efficiency. Ecotoxicology.

[CR11] Fountain M, Cross JV, Jåstad G, Hall DR, Douglas P, Farman DI (2014). Further studies on sex pheromones of female *Lygus* and related bugs (Heteroptera: Miridae): development of effective lures and investigation of species-specificity. J Chem Ecol.

[CR12] Francke W, Lübke G, Schröder W, Reckziegel A, Imperatriz-Fonseca V, Kleinert A, Engels E, Hartfelder K. Radtke R, Engels W. (2000) Identification of oxygen containing volatiles in cephalic secretions of workers of Brazilian stingless bees. J Brazil Chem Soc 11:562–571

[CR13] Gemeno C, Hall D, Guerrero A, Riba M, Cabrerizo A, Alomar O, Castañe C (2006). Potential sex pheromone components of the plant bug *Macrolophus caliginosus*. 22nd annual meeting. International Society of Chemical Ecology. Barcelona, Spain, July 15-19. Poster presentation

[CR14] Jacobson R (2019) *Nesidiocoris tenuis* biology and identification AHDB Factsheet 03/17

[CR15] Jarau S, Dambacher J, Twele R, Aguilar I, Francke W, Ayasse M (2010). The trail pheromone of a stingless bee, *Trigona corvina* (Hymenoptera, Apidae, Meliponini), varies between populations. Chem Senses.

[CR16] Kakizaki M, Sugie HJ (2001) Identification of female sex pheromone of the rice leaf bug, *Trigonotylus caelestialium*. J Chem Ecol 27:2447–2458 10.1023/a:1013623414324 11789951

[CR17] Kim JG, Lee WH, Yu YM, Yasunaga-Aoki C, Jung SH (2016). Lifecycle, biology, and descriptions of greenhouse biological control agent, *Nesidiocoris tenui*s (Reuter, 1895) (Hemiptera: Miridae). J Fac Agr Kyushu U.

[CR18] Lenth R (2019). Emmeans: estimated marginal means, aka least-squares means. R package version 1.3.4. https://CRAN.R-project.org/package=emmeans

[CR19] Lichtenberg EM, Hrncir M, Turatti IC, Nieh JC (2011) Olfactory eavesdropping between two competing stingless bee species. Behav Ecol Sociobiol 65:763–774 10.1007/s00265-010-1080-3 PMC3058493 21475736

[CR20] Mahob RJ, Babin R, ten Hoopen M, Dubog L, Yede Hall DR, Bilong Bilong CF (2011). Field evaluation of synthetic sex pheromone traps for the cocoa mirid *Sahlbergella singularis* (Hemiptera: Miridae). Pest Manag Sci.

[CR21] Mahot HC, Mahob RJ, Hall DR, Arnold SEJ, Fotso KA, Membang G, Ewane N, Kemga A, Fiaboe KKM, Bilong BCF, Hanna R (2020). Trap colour affects catches of brown cocoa mirid, *Sahlbergella singularis* Haglund, in sex pheromone traps in Cameroon cocoa plantations. Crop Prot.

[CR22] Nakaishi K, Fukui Y, Arakawa R (2011). Reproduction of *Nesidiocoris tenuis* (Reuter) on sesame. Japan J Appl Entomol Z.

[CR23] Neises B, Steglich W (1978). Simple method for the esterification of carboxylic acids. Angew Chem Int Edit.

[CR24] Padi B, Oduor G, Hall DR (2002) Development of mycoinsecticides and pheromones for cocoa mirids in Ghana. UK Department for International Development Final Technical Report l October 1998–31 March 2002. pp. 45. https://assets.publishing.service.gov.uk/media/57a08d38e5274a31e000170c/R7249_FTR.pdf

[CR25] PAN Europe, 2013 URL: https://www.pan-europe.info/old/Resources/Reports/PANE - 2013 - reducing pesticide use across the EU.Pdf

[CR26] Pérez-Hedo M, Urbaneja A (2016) The zoophytophagous predator *Nesidiocoris tenuis*: a successful but controversial biocontrol agent in tomato crops. In: Horowitz A., Ishaaya I. (eds) Advances in insect control and resistance management. Springer

[CR27] Pérez-Hedo M, Rambla JL, Granell A, Alberto Urbaneja A (2018). Biological activity and specificity of Miridae-induced plant volatiles. BioControl.

[CR28] Pérez-Hedo M, Riahi C, Urbaneja A (2021). Use of zoophytophagous mirid bugs in horticultural crops: current challenges and future perspectives. Pest Manag Sci.

[CR29] Raman K, Sanjayan KP (1984). Histology and histopathology of the feeding lesions by *Cyrtopeltis tenuis* rent. (Hemiptera: Miridae) on *Lycopersicon esculentum* mill. (Solanaceae). Proc Anim Sci.

[CR30] Sanchez AJ (2008). Zoophytophagy in the plantbug *Nesidiocoris tenuis*. Agr Forest Entomol.

[CR31] Sarfo JE (2013) Behavioural responses of cocoa mirids, *Sahlbergella singularis* Hagl. And *Distantiella theobroma* Dist. (Heteroptera: Miridae) to sex pheromones. Thesis submitted to the University of Greenwich

[CR32] Sarfo JE, Campbell CAM, Hall DR (2018). Design and placement of synthetic sex pheromone traps for cacao mirids in Ghana. Int J Trop Insect Sci.

[CR33] Sarfo JE, Campbell CAM, Hall DR (2018). Optimal pheromone trap density for mass trapping cacao mirids. Entomol Exp Appl.

[CR34] Staddon BW (1986). Biology of scent glands in the Hemiptera: Heteroptera. Ann Soc Entomol Fr.

[CR35] Urbaneja A, Tapia G, Stansly P (2005). Influence of host plant and prey availability on developmental time and survivorship of *Nesidiocoris tenuis* (Het.: Miridae). Biocontrol Sci Techn.

[CR36] Zhang T, Mei X, Zhang X, Lu Y, Ning J, Wu K (2020). Identification and field evaluation of the sex pheromone of *Apolygus lucorum* (Hemiptera: Miridae) in China. Pest Manag Sci.

