# Supplementary material for: Hero Turned Villain: Identification of Components of the Sex Pheromone of the Tomato Bug, Nesidiocoris tenuis
Source: J Chem Ecol. 2021 Apr 12;47(4):394–405. doi: 10.1007/s10886-021-01270-1 (PMC8116296; doi:10.1007/s10886-021-01270-1)
Supplement: Supplementary file 1 — (DOCX 112 kb) [file 10886_2021_1270_MOESM1_ESM.docx]

# Supplementary Material

# Hero turned Villain: Identification of Components of the Sex Pheromone of the Tomato Bug, *Nesidiocoris tenuis*

### David R Hall^1^ Steven J Harte^1^ Daniel P Bray^1^ Dudley I Farman^1^ Rob James^2^ Celine X Silva^3^ Michelle T Fountain^3^

^1^ Natural Resources Institute, University of Greenwich, Chatham Maritime, Kent, UK

^2^ Thanet Earth Ltd, Barrow Man Road, Birchington, Kent, UK

^3^ NIAB EMR, New Road, East Malling, Kent, UK

## Synthesis

### Octyl Hexanoate (II)

1-Octanol (2.6 g; 20 mM), hexanoic acid (2.32 g; 20 mM) and 4-dimethylaminopyridine (0.122 g; 1 mM) were dissolved in anhydrous dichloromethane (25 ml) and N,N’-dicyclohexylcarbodiimide (4.53 g; 22 mM) was added portion-wise over 30 min with stirring at room temperature. After stirring for 3 h, petroleum spirit (b.p. 40-60°C; 25 ml) was added. The mixture was filtered to remove most of the N,N’-dicyclohexylurea, washed with brine containing a little 2 N H2SO4, then with brine containing a little 2N KOH, and dried with anhydrous magnesium sulfate. Solvents were removed on a rotary evaporator and the residue was chromatographed on silica gel (35-70 µm; 100 g; Fisher) eluted with 2% diethyl ether in petroleum spirit. The product was distilled in a kugelrohr apparatus at 100 °C/0.06 mm Hg (4.1 g; 90%). ^1^H NMR (500 MHz; CDCl_3_) 4.06 t J=7.0 2H; 2.29 t J=7.5 2H, 1.62 m 4H, 1.3 m 14H; 0.90 t J=7; 0.88 t J=7 6H; ^13^C NMR (125 MHz; CDCl_3_) 13.926, 14.093, 22.339, 22.649, 24.724, 25.940, 28.658, 29.197, 29.216, 31.338, 31.787, 34.391, 64.413, 174.037; Mass spectrum Fig. S1.

### Hexyl Octanoate (III)

Hexyl octanoate was prepared similarly from 1-hexanol and octanoic acid in 93% yield. ^1^H NMR (500 MHz; CDCl_3_) 4.06 t J=7.0 Hz 2H; 2.29 t J=7.5 2H; 1.62 m 4H; 1.29 m 14H; 0.89 t J=7.0; 0.88 t J=7 6H; ^13^C NMR (125 MHz; CDCl_3_) 14.007, 14.074, 22.558, 22.610, 25.047, 25.620, 28.634, 28,949, 29.130, 31.452, 31.686, 34.428, 64.407, 174,041; Mass spectrum Fig. S1.


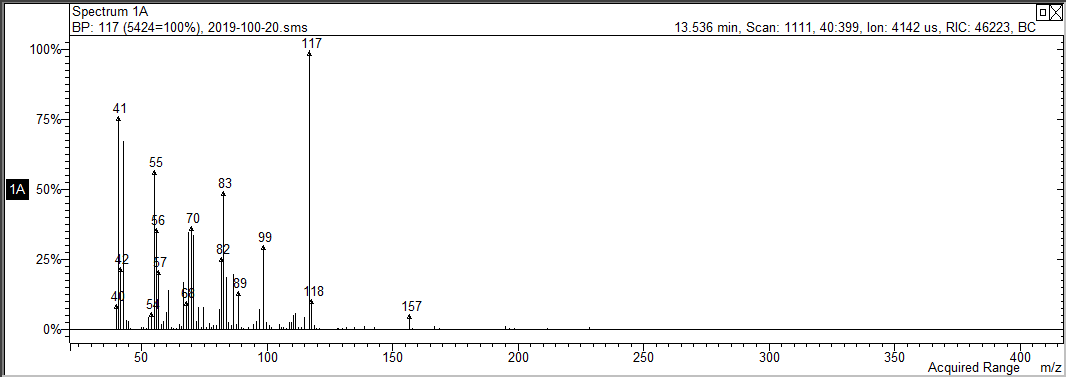


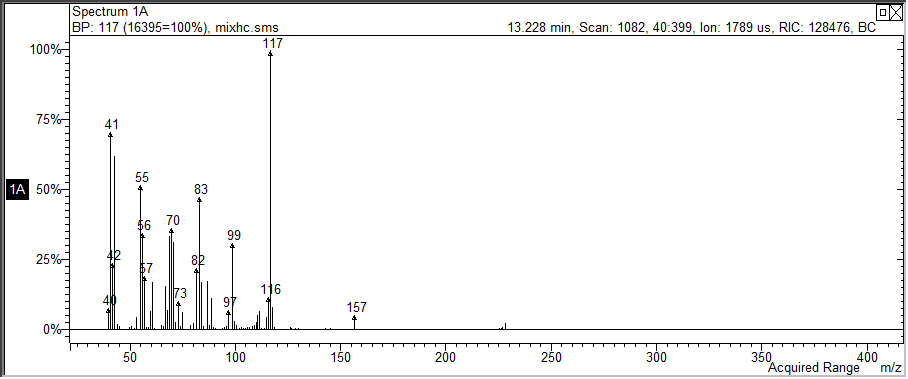


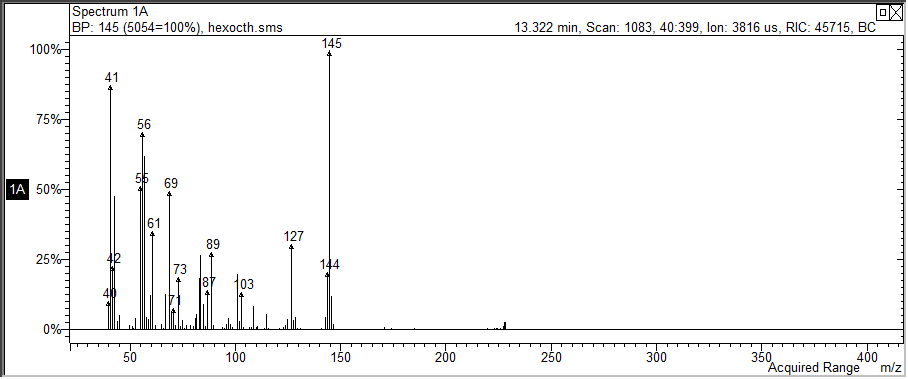


**Fig. S1** Mass spectra of pheromone component (B) (upper) octyl hexanoate (II) (middle) and hexyl octanoate (III) (lower)


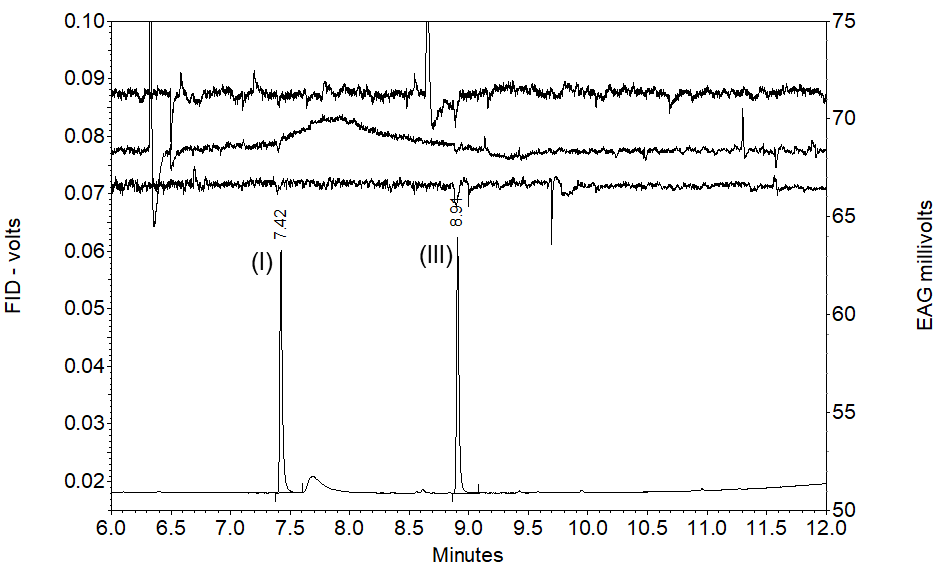


**Fig. S2**  GC-EAG Analyses of synthetic compounds (20 ng injected) with male *Nesidiocoris tenuis* antennal EAG preparation on polar GC column showing EAG responses to 1-octanol (I) at 7.42 min and the pheromone analog, hexyl octanoate (III) at 8.91 min
